# COVID-19 Incidence and Vaccine Effectiveness in University Staff, 1 March 2020–2 April 2022

**DOI:** 10.3390/vaccines11020483

**Published:** 2023-02-19

**Authors:** Luca Cegolon, Corrado Negro, Marco Pesce, Francesca Larese Filon

**Affiliations:** 1 Department of Medical, Surgical & Health Sciences, University of Trieste, 34129 Trieste, Italy; 2 Clinical Unit of Occupational Medicine, Department of Medical, Surgical & Health Sciences, University of Trieste, 34129 Trieste, Italy

**Keywords:** SARS-CoV-2, COVID-19, incidence, vaccine effectiveness, booster dose, university staff, healthcare workers

## Abstract

**Background**: University workers undergo intense social interactions due to frequent contact with students and colleagues and lectures in crowdy conditions. The aim of our study was to assess the incidence of COVID-19 infection and vaccine effectiveness in a cohort of workers of the University of Trieste from 1 March 2020 (start of the pandemic) through 2 April 2022. **Methods:** The University of Trieste implemented a number of public health policies to contain the spread of SARS-CoV-2 on the campus, including prompt contact tracing, enhanced ventilation of all premises, fomites disinfection and mandatory use of face masks indoors. In compliance with the surveillance protocol of the local public health department, university personnel were tested for SARS-CoV-2 by polymerase chain reaction (PCR) on a nasopharyngeal swab on demand, in the event of symptoms consistent with COVID-19 or for contact tracing, following close contact with a confirmed COVID-19 case. The incidence rates of SARS-CoV-2 infections were estimated as number of cases by number of person-days (p-d) at risk. Multivariable Cox proportional hazard regression model was employed to investigate the risk of primary COVID-19 infection, controlling for a number of potential confounders and expressing the risk as the adjusted hazard ratio (aHR) with a 95% confidence interval (95% CI). **Results:** The incidence of SARS-CoV-2 infection among university staff was lower than that of healthcare workers (HCWs) of the same area. Compared to unvaccinated colleagues (6.55 × 10,000 p-d), the raw incidence of SARS-CoV-2 infection was higher among university workers immunized with one (7.22 × 10,000 p-d) or two (7.48 × 10,000 p-d) doses of COVID-19 vaccines, decreasing in those receiving the booster (1.98 × 1000 p-d). The risk of infection increased only in postgraduate medical trainees (aHR = 2.16; 95% CI: 1.04; 4.48), though this was limited to the Omicron transmission period. After the implementation of the national vaccination campaign against COVID-19, workers immunized with the booster were less likely than unvaccinated workers to be infected by SARS-CoV-2 both before (aHR = 0.10; 95% CI: 0.06; 0.16) and after (aHR = 0.37; 95% CI: 0.27; 0.52) the Omicron transmission period. Vaccine effectiveness of the booster was 90% (=(1−0.10) × 100) before versus 63% (=(1−0.37) × 100) during the Omicron wave, without a significant difference between homologous (three doses of m-RNA vaccines) and heterologous immunization (first two doses of Vaxzevria followed by a third dose of m-RNA vaccine). **Conclusions:** The incidence of SARS-CoV-2 infection in university staff was lower than that of HCWs of ASUGI, likely because the testing-on-demand schedule inevitably missed the vast majority of asymptomatic infections. Therefore, the observed significantly protective effect of the booster dose in university personnel referred to symptomatic SARS-CoV-2 infections. The infection prevention and control policies implemented by the University of Trieste managed to equalize the biological risk between administrative and teaching staff.

## 1. Background

Since the start of the pandemic, epidemiological investigations on COVID-19 have focused on healthcare workers (HCWs) due to their elevated biological risk, high COVID-19 vaccination coverage and routine yet mandatory screening for SARS-CoV-2 infection [1,2,3,4,5,6].

However, despite health protection protocols, any indoor work activity involving a close proximity to others and contact with the public can facilitate SARS-CoV-2 infection, potentially even more than in the high-risk healthcare sector [7]. For instance, in a phone-based non-probability interview on 451 (13%) out of 3475 potentially eligible California workers tested for SARS-CoV-2 between November 2020 and March 2021, 212 positive versus 239 negative occupations were categorized by a SARS-CoV-2 job exposure matrix, three exposure metrics and a combination index. Study subjects working in proximity with others and with the highest combined exposure index were more likely to be infected by SARS-CoV-2 in the latter study [7].

An association between COVID-19 infection and occupational exposure, as assessed by the Mat-O-Covid job exposure matrix, was reported in 18,999 subjects with 389 different jobs, with the proportion of infection attributable to work estimated to range between 20% and 40% [8]. Likewise, occupational COVID-19 during the first pandemic wave in Italy accounted for 30% of cases [9]. In Belgium, during autumn 2020, business sectors defined by the Jobs at Risk Index (JARI) as occupations with only close proximity, without regular contact with the disease (education, law enforcement, fitness, beauty, retail, musicians/actors, restaurants and bars and transport), all had an equally high elevated incidence [10,11].

Educational staff undergo intense social interactions due to frequent contact with students and colleagues and lectures in crowdy conditions. Moreover, in addition to teaching, academic clinicians also have contact with patients; hence, their biological risk is comparable to full-time HCWs [12]. In fact, in Belgium, during autumn 2020, regional education workers reportedly had a higher incidence of COVID-19 than HCWs [10].

Since epidemiological data on academic personnel are missing in Italy, this study aims to investigate the risk of COVID-19 and associated factors in staff of the University of Trieste (Friuli Venezia Giulia Region, North-Eastern Italy) from the start of the pandemic in Italy (1 March 2020) through April 2022, contrasting the latter figures with those of the general population and of HCWs of the same geographical area.

## 2. Materials & Methods

### 2.1. Ethical Considerations

This study was approved by the regional ethics committee (CEUR) of Friuli-Venezia Giulia Region (Reg N.H32/2021). In compliance with Italian legislation on privacy law, informed consent from the study participants was waived since patients’ data were routinely collected for healthcare purposes and were managed anonymously within the framework of an approved study protocol. This study followed the Strengthening of Observational Studies in Epidemiology (STROBE) reporting guidelines.

### 2.2. Study Population

This study investigated the incidence of COVID-19 in 2323 workers of the University of Trieste, residing within the catchment area of the University Health Agency Giuliano-Isontina (ASUGI), covering the provinces of Trieste as well as Gorizia (Friuli-Venezia Giulia region), from 1 March 2020 through 2 April 2022.

### 2.3. Data Collection

The cohort included workers employed by the University of Trieste on a temporary or permanent contract. Staff hired as occasional teaching experts were excluded. Information on socio-demographic profiles (sex, age, occupation, department), number of doses of COVID-19 vaccines received, type of vaccine administered, total number of swab tests performed and dates of positive swab tests was available.

In compliance with the surveillance protocol of the local public health department of ASUGI, university workers underwent polymerase chain reaction (PCR) on a nasopharyngeal swab in the event of symptoms consistent with COVID-19 or following close contact with a confirmed COVID-19 case.

### 2.4. Statistical Analysis

Anonymized health surveillance data were stored in an Excel file and analyzed with STATA 16.0 (StataCorp LLC, College Station, TX, USA). Continuous data were reported as mean ± standard deviation (SD) and contrasted by *t*-test for normally distributed variables. Categorial variables were expressed as numbers and percentages and compared by chi square test.

The incidence rates of SARS-CoV-2 infections were estimated as number of events by person-days (p-d) at risk.

Multivariable Cox proportional hazard regression model was employed to investigate the risk of primary SARS-CoV-2 infections by explanatory factors displayed in Table 1. Since the national vaccination campaign against COVID-19 started officially on 27 December 2020 in Italy, in order to disentangle the impact of the pandemic on different periods, three multiple regression models were fitted separately to investigate the risk of primary SARS-CoV-2 infections, as follows:

- Pre-vaccination era: 1 March 2020–26 December 2020;
- Post-vaccination era, before the spread of the Omicron variant: 27 December 2020–30 November 2021;
- During the Omicron transmission period: 1 December 2021–2 April 2022.

Lastly, the risk of primary SARS-CoV-2 infection was compared during the Omicron transmission period (1 December 2021–2 April 2022) between those immunized by homologous vaccination—three doses of m-RNA vaccines (either Spikevax or Comirnaty)—and those immunized by heterologous vaccination—first two doses of Vaxzevria, followed by an m-RNA vaccine as a booster (either Spikevax or Comirnaty).

The results were expressed as adjusted hazard ratio (aHR) with 95% confidence intervals (95% CI). Vaccine effectiveness (VE = (1-aHR) × 100) against primary SARS-CoV-2 infections was calculated by number of doses of COVID-19 vaccines received 7 + days (14 + days in cases of only one dose) before SARS-CoV-2 infection.

Missing values were excluded, and complete case analysis was performed.

## 3. Results

Table 1 shows the distribution of COVID-19 cases among 2323 employees of the University of Trieste from 1 March 2020 through 2 April 2022 (25 months) by explanatory factors. During the entire study period (1 March 2020 through 2 April 2022), the cumulative crude incidence of COVID-19 in workers of the University of Trieste was 27.4%, compared to 11.7% (=58,873/473,896) in the general population of ASUGI and 40.0% (=3109/7723) in HCWs of ASUGI.

Although clinical information on COVID-19 symptoms was not available, only one worker (vaccinated with three doses) was hospitalized for COVID-19 in October 2021 (during the Delta transmission period in Trieste). Therefore, the other workers developed a mild–moderate disease, not requiring hospital admission.

As can be noted from Table 1, the distribution by sex was balanced, and the average age of the study subjects was 47.9 ± 13.9 years. Workers infected by SARS-CoV-2 were slightly younger (44.9 ± 13.3 vs. 48.5 ± 14.0 years), and a decreasing rate of COVID-19 infections with age could be observed. The cohort was mainly composed of PhD students (27.7%), followed by administrative clerks (21.2%) and academic staff (21.2%). The most represented department was healthcare (31.5%), followed by administrative services (25.6%). The most used vaccine type for the first and second doses was Comirnaty (Pfizer BioNTech, 52.2% vs. 54.0%), followed by Vaxzevria (Oxford–Astrazeneca, 42.3% vs. 41.4%) and Spikevax (Moderna, 5.0% vs. 4.5%). By contrast, the most used vaccine type for the third dose was Comirnaty (47.2%), followed by Spikevax (35.6%). A total of 56.4% (=1052/1864) of workers received a homologous booster (three doses of m-RNA vaccines, either Comirnaty or Spikevax), compared to 43.6% (=812/1864) who were immunized with two doses of Vaxzevria followed by a heterologous booster (either Spikevax or Comirnaty).

**Figure 1:**
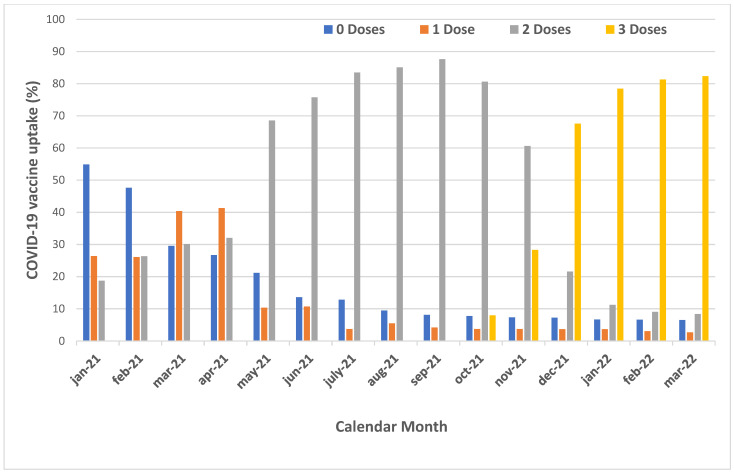
Cumulative uptake of the COVID-19 vaccination among workers of the University of Trieste over time, by number of doses of the COVID-19 vaccine.

Table 2 and Figure 1 show the vaccine uptake by number of doses, type of COVID-19 vaccine received and calendar month. COVID-19 vaccination coverage by 2 April 2022 (study end) was as follows: 82.4% (=1913 /2223) university workers were vaccinated with three doses, 8.4% (=194/2223) with two doses, 2.7% (=63/2223) were vaccinated with one dose and 3.1% (=71/2223) of HCWs were fully unvaccinated. University staff started to receive the booster by 4 September 2021, and 28.3% were immunized with three doses by 30 November 2021.

**Figure 2:**
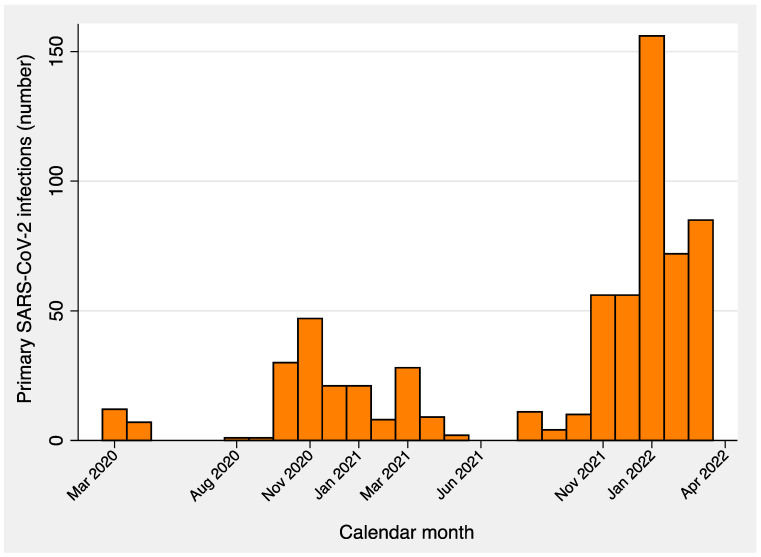
Distribution of primary SARS-CoV-2 infections by calendar month (1 March 2020–2 April 2022) among workers of the University of Trieste.

**Figure 3:**
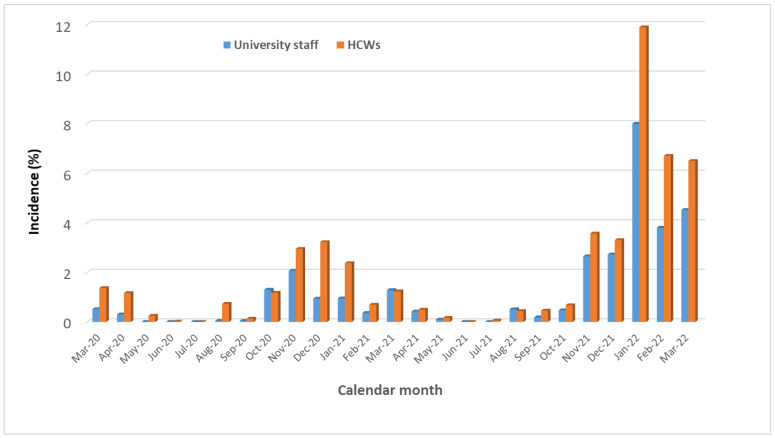
Monthly incidence (%) of SARS-CoV-2 infections in healthcare workers (HCWs) versus the university staff of Trieste.

Figure 2 shows the distribution of primary SARS-CoV-2 infections by calendar month, whereas Figure 3 contrasts the monthly incidence of COVID-19 among the staff of the University of Trieste with that of HCWs of ASUGI. Table 3 displays the distribution of primary SARS-CoV-2 infections by COVID-19 wave, pandemic era and number of doses of vaccines received 7 + days (14 + in cases of only one dose) before the infection. As can be seen, out of 637 primary SARS-CoV-2 infections recorded during the entire study period, 28 cases of re-infections were observed, all occurring from 11 December 2021 onward. The majority of COVID-19 cases surged from November 2021 on, peaking in January 2022, during the Omicron transmission period. In particular, 129 COVID-19 cases were notified before the vaccination era, 139 were recorded in the post-vaccination era before the spread of the Omicron variant, and 369 were recorded from 1 December 2021 through 2 April 2022, during the Omicron transmission period. During October–November 2021, when the immunization campaign for the third dose started in Trieste, 66 COVID-19 cases were recorded, against a booster uptake of 28.3% by 30 November 2021.

Table 4 shows the distribution of primary SARS-CoV-2 infections by number of doses of vaccine received 7 + days (14 + days in cases of first dose) before the infection, person-days (p-d) at risk by each stratum and the respective crude incidence rate x 10,000 p-d. As can be seen, in the entire period (1 March 2020–2 April 2022), the crude incidence of unvaccinated staff was 6.55 × 10,000 p-d, compared to 3.77 × 10,000 p-d among the entire cohort of workers (also including vaccinated individuals). Compared to unvaccinated, the raw incidence of SARS-CoV-2 infection was higher in those immunized with one (7.22 × 10,000 p-d) or two (7.48 × 10,000 p-d) doses, decreasing in workers receiving the booster (1.98 × 10,000 p-d).

After the start of the administration of the third COVID-19 vaccine dose (4 September 2021), the incidence rate of SARS-CoV-2 infection decreased to nil for those immunized with a heterologous booster (first two doses of Vaxzevria, followed by one dose of an m-RNA vaccine), compared to 0.05 × 10,000 p-d among those receiving three doses of m-RNA vaccines (homolgous booster).

During the Omicron transmission period (1 December 2021–2 April 2022), the crude incidence rate of infection increased, though it was still higher among workers immunized by homologous (2.43 × 10,000 p-d) versus heterologous (1.43 × 10,000 p-d) boosters.

Table 5 displays three multivariable Cox proportional regression models investigating the risk of primary SARS-CoV-2 infections by pandemic period (pre-vaccination era, pre-Omicron era and Omicron transmission period). As can be noted, the risk of SARS-CoV-2 infection was not associated with age, sex, occupation or university department in the pre-vaccination period. By contrast, after the implementation of the vaccination campaign against COVID-19, workers immunized with the booster were less likely than unvaccinated colleagues to be infected by SARS-CoV-2 both before (aHR = 0.10; 95% CI: 0.06; 0.16) and after (aHR = 0.37; 95% CI: 0.27; 0.52) the Omicron transmission period. This resulted in a VE of the booster equal to 90% ((=1−0.10) × 100) before versus 63% ((=1−0.37) × 100) during the Omicron wave.

Furthermore, the risk of infection during the Omicron transmission period was significantly higher for university workers aged 41–55 years (aHR = 2.59; 95% CI: 1.39; 4.83) and lower for those aged 56 + years (aHR = 0.63; 95% CI: 0.45; 0.87). Lastly, during the Omicron transmission period, postgraduate specialist medical trainees (aHR = 2.16; 95% CI: 1.04; 4.48) were more likely to be infected compared to administrative clerks.

**Figure 4:**
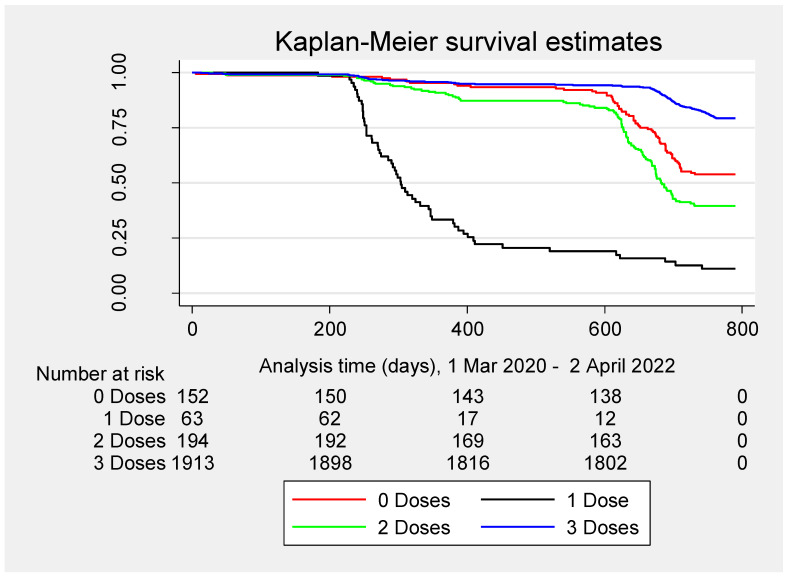
Kaplan–Meier survival curve for the risk of primary SARS-CoV-2 infections by the number of doses of COVID-19 vaccines, regardless of whether they preceded primary SARS-CoV-2 infections.

Figure 4 shows the Kalan–Meier curve for the incidence of primary SARS-CoV-2 infections by the number of doses of COVID-19 vaccines received during the entire period (1 March 2020–2 April 2022), regardless of whether they preceded primary SARS-CoV-2 infections.

Table 6 displays the multivariable Cox proportional regression analysis investigating the risk of primary SARS-CoV-2 infections during the Omicron transmission period (1 December 2021–2 April 2022) by heterologous vs. homologous vaccination, adjusting for potential confounders. Only significant stratum-specific estimates are displayed. As can be seen, the risk of primary SARS-CoV-2 infection in those immunized with a homologous booster was significantly higher in the crude analysis (HR = 1.76; 95% CI: 1.37; 2.27) and was confirmed in the model adjusted for sex (aHR = 1.76; 95% CI: 1.36; 2.27), as well as that adjusted for sex plus age (aHR = 1.54; 95% CI: 1.18; 2.02). However, the latter difference waned after controlling for the effect of the university department and job task.

## 4. Discussion

### 4.1. Key Findings

By 1 December 2021, 28.3% of workers at the University of Trieste had received the booster, compared to 60.6% who were immunized with two doses of the COVID-19 vaccine. Vaccine uptake of the booster progressively increased over time, reaching 78.4% by January 2022 and 82.4% by the study’s end (2 April 2022). A total of 56.4% of workers received a homologous booster, compared to 43.6% who were immunized with a heterologous combination.

Six hundred and thirty-seven primary SARS-CoV-2 infections were recorded among the personnel of the University of Trieste during the entire study period, lasting 25 months (1 March 2020–2 April 2022). The majority (57.4%) of primary infections were recorded from 1 December 2021 onward, during the Omicron transmission period. Likewise, 28 cases of re-infections were observed only from 1 December 2021 onward. By contrast, the number of primary SARS-CoV-2 infections in the pre-vaccination (N = 129) and post-vaccination periods preceding the Omicron wave (N = 139) was similar.

During the entire study period (1 March 2020 through 2 April 2022), the cumulative crude incidence of COVID-19 among workers of the University of Trieste was 27.4%, compared to 11.7% among the general population of ASUGI and 40.0% among HCWs of ASUGI. Only one university worker (immunized with the booster) was hospitalized for COVID-19 (in October 2021, during the Delta wave), whereas the remaining developed only a mild–moderate disease, not requiring hospitalization.

The main factor associated with SARS-CoV-2 infection was COVID-19 vaccination status before SARS-CoV-2 infection. In particular, compared with unvaccinated colleagues, the risk of infection decreased significantly in workers immunized with the booster, whose VE was 90% before the spread of the Omicron variant, decreasing to 63% afterwards. The raw incidence rate of SARS-CoV-2 infection was nil or almost nil during the pre-Omicron wave for homologous and heterologous booster doses, respectively. Although the crude infection risk slightly increased during the Omicron wave among those immunized with three doses, being higher for homologous versus heterologous immunization, the latter difference disappeared after adjusting for the effects of university department and job task.

The only occupational category at a higher risk for SARS-CoV-2 infection was postgraduate medical trainees, though this was limited to the Omicron transmission period.

During the Omicron wave, older university workers (>55 years of age) were less likely to be infected, whereas in the 11 months preceding the Omicron wave, individuals aged 41–55 were at higher biological risk.

### 4.2. Interpretation of the Findings

The cohort of workers of the University of Trieste comprised academic staff (professors and researchers), administrative clerks and technicians from 10 different departments, including healthcare.

Following the country lockdown in Italy (31 May 2020), in order to resume in-person lessons and research activities as soon as possible, the University of Trieste implemented a number of preventative measures to contain SARS-CoV-2 transmission in academic settings, enforcing social distancing and systematic fomites disinfection and encouraging staff and students to engage in frequent hand washing. Face masks were made mandatory for all individuals in indoor places on the campus until the end of September 2022. Smart-working and remote lectures were maintained for vulnerable workers with an individual susceptibility to infections. Since air quality of indoor public places impacts the spread of COVID-19, all premises of the University of Trieste were subject to enhanced ventilation and low-cost sensors for intelligent monitoring of indoor CO_2_, were considered to mitigate the spread of SARS-CoV-2 and prevent common symptoms such as coughs, headaches, eye irritation, dizziness and fatigue [13].

Furthermore, systematic and intense contact tracing was implemented among university staff as well as students for early detection of SARS-Cov-2 infections, in compliance with Italian law. A COVID-19 taskforce was set up by the unit of occupational medicine of ASUGI to assess the weekly number of COVID-19 incident cases and the effectiveness of the infection prevention and control measures enforced in university settings.

The higher incidence of COVID-19 among workers of the University of Trieste compared to the general population of the same area is likely attributable to more intense contact tracing, especially for academic healthcare staff, characterized by an intrinsically higher occupational exposure to SARS-CoV-2.

By contrast, the cumulative incidence of COVID-19 among the personnel of the University of Trieste (27.4%) was much lower than that of HCWs of ASUGI, whose incidence was 46.1% (= 3561/7723) during 1 March 2020–31 May 2022, a figure slightly reduced to 39.5% by restricting the observation time to the same period as that of the present study (1 March 2020–2 April 2022). The latter difference widens further considering p-d at risk as a denominator between the two cohorts, with a crude incidence of 97 × 10,000 p-d for HCWs (during 1 Dec 2021–31 May 2022) compared to 3.77 × 10,000 p-d for university staff (during 1 March 2020–2 April 2022). The latter discrepancy is likely attributable to asymptomatic SARS-CoV-2 infections, which could be captured by a mandatory routine testing schedule for HCWs but were inevitably missed by testing-on-demand schedule of university personnel.

Likewise, the only occupational category at higher risk of SARS-CoV-2 infection in the present study was doctors in postgraduate specialist training, albeit only during the Omicron transmission period. The risk of COVID-19 is usually higher for HCWs compared with other occupations; this is a result of higher occupational risk and routine testing schedule [14,15,16,17,18]. Furthermore, since they are typically younger, trainee doctors were assigned to front-line job tasks since the early stage of the pandemic due to the lower susceptibility to developing severe COVID-19. Moreover, young trainee doctors normally have more intense social interactions outside work, are less keen to comply with non-pharmaceutical risk reduction measures outside the workplace and frequently share living places.

The reduced risk of COVID-19 among older university workers during the Omicron transmission period in the present study might reflect higher level of compliance with non-pharmaceutical risk reduction measures. By contrast, the increased risk of COVID-19 among university staff aged 41–55 years during the pre-Omicron period might be attributable to the high-risk contact after the start of the COVID-19 vaccination campaign and the resumption of in-person academic activities.

Apart from postgraduate medical trainees and unspecified university departments, no other occupational factor was significantly associated with SARS-CoV-2 infection in the present study. The latter figures may be attributable to the effectiveness of the non-pharmaceutical risk reduction strategies implemented by the University of Trieste to prevent the spread of SARS-CoV-2 during learning activities, equalizing the risk of infection between administrative clerks and teaching staff. Moreover, during the first pandemic year, university activities were allowed only by distance. This could explain why no occupational factor increased the risk of COVID-19 during the pre-vaccination era in the present study, when several business activities were suspended in Italy, especially during the country’s lockdown, thereby minimizing social interactions.

### 4.3. Vaccine Effectiveness

We found a booster VE of 90% before the Omicron transmission period, which was reduced to 63% from December 2021 onward. The raw incidence rate of SARS-CoV-2 infection was nil or almost nil during the pre-Omicron wave for homologous and heterologous booster doses, respectively. Although the crude infection risk slightly increased during the Omicron wave, being higher for homologous versus heterologous immunization, the latter difference disappeared after adjusting for the prevailing effect of university department and job task.

In a previous study on HCWs from the same area, VE of the second as well as the third doses of the COVID-19 vaccine vanished during the Omicron transmission period. Again, the latter finding can be explained by different testing schedules between HCWs and university personnel. HCWs are characterized by a high biological risk and, depending on their occupational exposure to COVID-19, are mandated to test weekly or monthly, a schedule that is also able to detect asymptomatic SARS-CoV-2 infections, which account for almost half of COVID-19 cases, especially during the Omicron transmission period [5,6,19,20]. By contrast, the university staff were tested on demand in the event of symptoms consistent with COVID-19 or for contact tracing in the event of exposure to a confirmed COVID-19 case. The latter approach inevitably misses most asymptomatic infections and, arguably, also a variable proportion of patients with flu-like symptoms. Therefore, the effectiveness of the booster in the preset study most likely reflected the protection against symptomatic COVID-19, in line with reports from phase 3 clinical trials of the respective COVID-19 vaccines [21,22,23]. Human coronaviruses can re-infect regardless of humoral immunity due to the high tendency of mutation, allowing them to evade neutralizing antibody responses [24,25]. All 28 cases of re-infections in the present study occurred from 1 December 2021 onward, with the spread of the Omicron variant, whose spike protein highly diverges from previous SARS-CoV-2 strains. The risk of vaccine failure also increased dramatically among vaccinated individuals from December 2021 onward, when Omicron spread globally, progressively replacing the Delta variant and peaking in January 2022 [5,6,20,26,27,28].

### 4.4. Generalizability

As already mentioned, while HCWs have been over-studied during the COVID-19 pandemic [1,2,3,4,5,6], epidemiological data on other occupational categories are scanty or totally missing, especially in Italy.

A few studies investigated COVID-19 in essential occupations over a limited timeframe [12,14]. Sixty-one clusters of COVID-19 were reported in Japan during 2020, not only among HCWs (30%) and other healthcare facilities (16%) but also in cultural activities (11%), gyms (8%), ceremonies (3%) and transport (3%) [29]. In the US and Canada, COVID-19 outbreaks were associated with industries, agriculture, forestry, fishing, hunting sectors, transportation and warehousing [30,31]. In Belgium, during autumn of 2020, a higher infection risk was reported not only for healthcare but also for food service, cultural and sport activities [10]. Further occupational sectors at higher risk of COVID-19 than the general population include food processing companies, bartenders, restaurants and bus, tram and taxi drivers [15,32,33,34,35].

A study on more than 100,000 workers during the first pandemic wave in Germany reported an increased risk of COVID-19 in essential occupations such as healthcare, logistics, transport, police, jurisdiction and public administration and in higher-occupational-status positions (e.g., managers and highly skilled workers) [13].

Another study designed to be representative of the UK general population investigated the occupational risk of COVID-19 using data from the UK Office of National Statistics COVID-19 Infection Survey from April 2020 through November 2021. The latter study, using regular PCR testing on 3,910,311 observations (visits) from 312,304 adults of working age, reported a higher occupational risk of SARS-CoV-2 infection in relation to social care (HR = 1.14; 95% CI: 1.04; 1.24), education (HR = 1.31; 95% CI: 1.23; 1.39), bus drivers (HR = 1.43; 95% CI: 1.03: 1.97) and police and protective services (HR = 1.45; 95% CI: 1.29; 1.62) compared to non-essential workers [14]. Following the first two pandemic waves, the risk of infection in the UK decreased only among HCWs, probably due to higher compliance with health protection measures, whereas it remained high for education staff, calling for long-term risk reduction strategies such as the forced indoor ventilation of teaching premises [14].

Pharmacological interventions, such as post-exposure prophylaxis, enhancing the physiological-specific defenses of upper airways against the entry of respiratory pathogens through the nasal cavity, may also be considered in the future to contain the spread of emerging SARS-CoV-2 variants in crowded indoor public places such as educational settings [19,24,36,37,38,39].

### 4.5. Strengths and Weaknesses

The present study is one of the few investigating the risk of SARS-CoV-2 infection among academic staff over a long period of time (25 months), confirming the higher biological risk associated with healthcare occupations.

In general, the validity of estimating the risk of COVID-19 across different occupations is debated, since different occupations entail different testing schedules and therefore different likelihoods of diagnosing SARS-CoV-2 infections. This potential bias does not only apply to HCWs. For instance, better-off individuals could be more likely to test for COVID-19 since they are less affected by the direct costs (testing fees) and financial backlashes due to a loss of income for sick leave in the event of testing positive for COVID-19 [14,40]. As already stressed, testing on demand inevitably missed asymptomatic infections. Furthermore, university personnel were allowed to work from home, especially during the first pandemic year.

Lastly, information on other potential cofounders that enhanced the risk of COVID-19 was not available.

## 5. Conclusions

The cumulative incidence of SARS-CoV-2 infections among university staff of Trieste was higher than that of the local general population and lower than that of HCWs from the same geographical area. The former figure likely reflects the effect of public health policies implemented by the University of Trieste for enhanced contact tracing. By contrast, the latter discrepancy is inevitably attributable to asymptomatic SARS-CoV-2 infections, which could be captured by the mandatory routine testing schedule of HCWs but were inevitably missed by testing on demand for university personnel.

The booster dose coverage among university staff was approximately 78.4% by January 2022, and workers who were unvaccinated or partially immunized faced work restrictions by Italian law (smart-working or even work suspension).

University workers immunized with the booster were less likely than unvaccinated colleagues to be infected by SARS-CoV-2 both before (VE = 90%) and during (VE = 63%) the Omicron transmission period. Since university workers were predominantly tested on demand, the latter protection is largely attributable to symptomatic infection. During the Omicron wave, there was no difference in the risk of primary SARS-CoV-2 infection between university staff immunized with three doses of m-RNA vaccines and those immunized by heterologous vaccination.

The infection prevention and control policies implemented by the University of Trieste, including prompt contact tracing, allowed for containment the containing of the spread of the infection in all academic settings, equalizing the biological risk between administrative and teaching personnel. Only postgraduate specialist medical trainees exhibited a higher occupational biological risk during the Omicron transmission period.

## Figures and Tables

**Table 1:** Distribution of primary COVID-19 cases by explanatory factors. Number (N), percentage (%) and chi-square *p*-value. M = missing values.

Terms			Total	Primary SARS-CoV-2 Infections		*p*-Value
				Negative	Positive	
**Total (row %)**			2323 (100)	1686 (72.6)	637 (27.4)	
**Sex**	**Females**		1140 (49.1)	827 (49.1)	313 (49.1)	0.971
	**Males**		1183 (50.9)	859 (50.9)	324 (50.8)	
**Age** (years)	**Mean ± SD**		47.9 ± 13.9	48.5 ± 14.0	44.9 ± 13.3	<0.001
	**<41**		766 (33.0)	516 (30.6)	250 (39.3)	<0.001
	**41–55**		759 (32.7)	537 (31.9)	222 (34.9)	
	**56+**		798 (34.5)	633 (37.5)	165 (25.9)	
**Occupation**	**Administrative Clerks**		493 (21.2)	394 (23.4)	144 (22.6)	<0.001
	**Academic staff**		493 (21.2)	388 (23.1)	105 (16.5)	
	**PhD students**		643 (27.7)	500 (29.7)	143 (22.5)	
	**Postgraduate specialist medical trainees**		213 (9.2)	113 (6.7)	100 (15.7)	
	**Short-term contract**	**Healthcare sector**	376 (16.2)	254 (15.1)	122 (19.2)	
		**Other**	60 (2.6)	37 (2.2)	23 (3.6)	
**Department**	**Administrative & technical support**		595 (25.6)	440 (26.10)	155 (24.3)	<0.001
	**Physics**		100 (4.3)	75 (4.5)	25 (3.9)	
	**Engineering & Architecture**		193 (8.3)	148 (8.8)	45 (7.1)	
	**Mathematics & Geosciences**		125 (5.4)	97 (5.8)	28 (4.4)	
	**Chemical & Pharmaceutical Sciences**		64 (2.8)	50 (3.0)	14 (2.2)	
	**Life Sciences**		168 (7.2)	134 (8.0)	34 (5.3)	
	**Economics, Business & Statistics**		66 (2.8)	54 (3.2)	12 (1.9)	
	**Law, Language & Interpreting**		75 (3.2)	62 (3.7)	13 (2.0)	
	**Political & Social Sciences**		34 (1.5)	29 (1.7)	5 (0.8)	
	**Human Sciences**		108 (4.7)	87 (5.2)	21 (3.30)	
	**Medical, Surgical & Health Sciences**		724 (31.2)	467 (27.1)	257 (40.4)	
	**Not specified**		71 (3.1)	43 (2.6)	28 (4.4)	
**N. doses of COVID-19 vaccine** (M: 19)	**0**		152 (6.5)	82 (4.9)	70 (11.0)	<0.001
	**1**		63 (2.7)	7 (0.42)	56 (8.8)	
	**2**		194 (8.4)	77 (4.6)	117 (18.5)	
	**3**		1913 (82.4)	1520 (90.2)	394 (61.9)	
	**4**		1	1	0	
**Total number of swab tests**	**Mean ± SD**		12.6 ± 15.8	10.1 ± 14.6	19.21 ± 17.0	<0.001
	**Median (IQR)**		4 (1; 23)	3 (1; 15)	12 (4; 33)	<0.001
	**0**		323 (13.9)	323	0	<0.001
	**1–2**		504 (21.7)	464 (27.5)	40 (6.3)	
	**3–5**		504 (21.7)	328 (19.5)	176 (27.6)	
	**6–26**		506 (21.8)	304 (18.0)	202 (31.7)	
	**27+**		486 (20.9)	267 (15.8)	219 (34.4)	
**First Vaccine dose** (M:19)	**Comirnaty**		1127 (52.2)	781 (48.9)	346 (61.7)	<0.001
	**Spikevax**		107 (5.0)	62 (3.9)	45 (8.0)	
	**Vaxzevria**		913 (42.3)	746 (46.7)	167 (29.8)	
	**Jannsen (N = 9)/Nuvaxovid (N = 2)**		11 (0.5)	8 (0.5)	3 (0.5)	
**Second Vaccine dose** (M:19)	**Comirnaty**		1130 (54.0)	803 (50.6)	327 (64.9)	<0.001
	**Spikevax**		94 (4.5)	63 (4.0)	31 (6.2)	
	**Vaxzevria**		865 (41.4)	719 (45.3)	146 (29.0)	
	**Nuvaxovid**		3 (0.1)	3 (0.2)	0	
**Third Vaccine dose** (M:19)	**Comirnaty**		1087 (47.2)	805 (53.2)	282 (71.6)	<0.001
	**Spikevax**		819 (35.6)	707 (46.7)	112 (28.4)	
	**Nuvaxovid**		1	1 (0.1)	0	
**Booster** **dose**	Heterologous *		812 (43.6)	705 (47.5)	107 (29.1)	<0.001
	Homologous **		1052 (56.4)	778 (52.5)	274 (71.9)	

* First two doses of Vaxzevria followed by a booster dose of m-RNA vaccine (either Comirnaty or Spikevax). ** First three doses of m-RNA vaccines (either Comirnaty or Spikevax).

**Table 2:** COVID-19 vaccination uptake among workers of the University of Trieste. Number (N) and row percentage (%).

Calendar Month	Vaccine Type	0 Doses	1 Dose	2 Doses	3 Doses	4 Doses
**27 Dec 2020–31 Jan 2021**	**Cumulative uptake**	**1275 (54.9)**	**613 (26.4)**	**435 (18.7)**	**0**	0
	**Comirnaty**		608	433		
	Unknown *		5	2		
**1–28 February 2021**	**Cumulative uptake**	**1106 (47.6)**	**606 (26.1)**	**611 (26.3)**	**0**	**0**
	**Comirnaty**		79	173		
	**Spikevax**		1	0		
	**Vaxzevria**		520	0		
	Unknown *		4	3		
**1–31 March 2021**	**Cumulative uptake**	**686 (29.5)**	**938 (40.4)**	**699 (30.1)**	**0**	0
	**Comirnaty**		39	82		
	**Spikevax**		1	1		
	**Vaxzevria**		380	0		
	Unknown *		0	5		
**1–30 April 2021**	**Cumulative uptake**	**620 (26.7)**	**959 (41.3)**	**744 (32.0)**	**0**	**0**
	**Comirnaty**		59	44		
	**Spikevax**		1	1		
	**Vaxzevria**		6	0		
**1–31 May 2021**	**Cumulative uptake**	**491 (21.1)**	**239 (10.3)**	**1593 (68.6)**	**0**	**0**
	**Comirnaty**		85	65		
	**Spikevax**		31	0		
	**Vaxzevria**		7	784		
	**Jannsen**		4	0		
	Unknown *		1	0		
**1–30 June 2021**	**Cumulative uptake**	**315 (13.6)**	**248 (10.7)**	**1760 (75.8)**	**0**	**0**
	**Comirnaty**		154	68		
	**Spikevax**		17	27		
	**Vaxzevria**		0	72		
	**Jannsen**		4	0		
	Unknown *		1	0		
**1–31 July 2021**	**Cumulative uptake**	**297 (12.8)**	**87 (3.7)**	**1939 (83.5)**	**0**	**0**
	**Comirnaty**		16	153		
	**Spikevax**		2	18		
	**Vaxzevria**		0	8		
**1–31 August 2021**	**Cumulative uptake**	**219 (9.4)**	**128 (5.5)**	**1976 (85.1)**	**0**	**0**
	**Comirnaty**		50	28		
	**Spikevax**		28	6		
	**Vaxzevria**		0	8		
	Unknown *		1	2		
**1–30 September 2021**	**Cumulative uptake**	**188 (8.1)**	**97 (4.2)**	**2035 (87.6)**	**2 (0.1)**	**0**
	**Comirnaty**		27	43	1	
	**Spikevax**		1	15	0	
	**Jannsen**		1	0	0	
	Unknown *		1	3	1	
**1–31 October 2021**	**Cumulative uptake**	**179 (7.7)**	**87 (3.7)**	**1873 (80.6)**	**184 (7.9)**	**0**
	**Comirnaty**		5	17	181	
	**Spikevax**		3	1	0	
**1–30 November 2021**	**Cumulative uptake**	**171 (7.4)**	**87 (3.7)**	**1408 (60.6)**	**657 (28.3)**	**0**
	**Comirnaty**		1	5	437	
	**Spikevax**		5	1	34	
	Unknown *		0	0	2	
**1–31 December 2021**	**Cumulative uptake**	**168 (7.2)**	**85 (3.7)**	**501 (21.6)**	**1569 (67.5)**	**0**
	**Comirnaty**		0	0	375	
	**Spikevax**		5	3	536	
	**Vaxzevria**		0	3	0	
	Unknown *		0	1	1	
**1–31 January 2022**	**Cumulative uptake**	**155 (6.7)**	**86 (3.7)**	**260 (11.2)**	**1822 (78.4)**	0
	**Comirnaty**		2	1	17	
	**Spikevax**		12	12	234	
	Unknown *		0	0	2	
**1–28 February 2022**	**Cumulative uptake**	**154 (6.6)**	**71 (3.1)**	**210 (9.0)**	**1888 (81.3)**	**0**
	**Comirnaty**		2	9	53	
	**Spikevax**		0	8	13	
**1 Mar 2022–2 Apr 2022**	**Cumulative uptake**	**152 (6.5)**	**63 (2.7)**	**194 (8.4)**	**1913 (82.4)**	**1**
	**Comirnaty**		0	6	23	0
	**Spikevax**		0	1	2	0
	**Nuvaxovid**		2	3	1	0
	Unknown *		0	0	0	1

* Vaccinations performed outside ASUGI, with only the date of immunization available.

**Table 3:** Distribution of SARS-CoV-2 infections by COVID-19 wave and number of vaccine doses received. Number (N) and column percentage (%).

SARS-CoV-2 Infections			Total (N = 637)
**COVID-19** **wave**		**I** (7 Mar 2020–31 May 2020)	19 (3.0)
		**II** (1 Jun 2020–30 Sep 2020)	2 (0.3)
		**III** (1 Oct 2020–31 Dec 2020)	98 (15.4)
		**IVa** (1 Jan 2021–31 Mar 2021)	57 (9.0)
		**IVb** (1 Apr 2021–30 Sep 2021)	26 (4.1)
		**V** (1 Oct 2021–30 Nov 2021)	66 (10.4)
		**VI** (1 Dec 2021–2 April 2022)	369 (57.9)
**Pandemic era**		**Pre-vaccination** (1 Mar 2020–09 Jan 2021)	129 (20.3)
		**Pre-Omicron** (10 Jan 2021–30 Nov 2021)	139 (21.8)
		**Omicron** (1 Dec 2021–2 Apr 2022)	369 (57.9)
**COVID-19** **vaccination**	**Before vaccination**		222 (34.9)
	**Between** **1st and 2nd dose**	**14 + days since 1st vaccine dose**	15 (2.4)
		**<14 days since 1st vaccine dose**	14 (2.2)
	**Between** **2nd and 3rd dose**	**7 + days since 2nd vaccine dose**	110 (17.3)
		**<7 days since 2nd vaccine dose**	0
	**Between** **3rd and 4th dose**	**7 + days since 3rd vaccine dose**	267 (41.9)
		**<7 days since 3rd vaccine dose**	9 (1.4)
**Re-infections**			28 (4.4)

**Table 4:** Crude incidence rates of primary SARS-CoV-2 infections during 1 March 2020–30 April 2022 by explanatory factors. Number of cases, person-days (p-d) at risk and raw incidence (×10,000 p-d).

Terms		Cases (Number)	Person-Days	Cases × 10,000 p-d
**Entire study period** **(1 Mar 2020–2 Apr 2022)**	**All workers**	637	1,690,346	3.77
	**Fully unvaccinated**	70	106,876.53	6.55
**Entire vaccination era** **(27 Dec 2020–2 Apr 2022)**	**14 + days since 1st dose**	7	9690.47	7.22
	**7 + days since 2nd dose**	89	118,917.79	7.48
	**7 + days since 3rd dose**	276	1,395,087	1.98
**Pre-Omicron wave** **(4 Sept 2021–30 Nov 2021)**	7 + days since homologous booster *	3	616,488	0.05
	7 + days since heterologous booster **	0	556,950	0
**Omicron wave** **(1 Dec 2021–2 April 2022)**	7 + days since homologous booster *	180	741,625.06	2.43
	7 + days since heterologous booster **	89	620,232.51	1.43

* Three doses of m-RNA vaccines (Spikevax or Comirnaty); ** first two doses of Vaxzevria, followed by a booster with an m-RNA vaccine (Spikevax or Comirnaty).

**Table 5:** Cox proportional regression model for the risk of primary SARS-CoV-2 infection (1 March 2020–30 April 2022) by pandemic period. **MODEL 1** (pre-vaccination era): 1 March 2020–26 December 2020; **MODEL 2** (pre-Omicron era): 27 December 2020–30 November 2021; **MODEL 3** (Omicron Transmission period): 1 December 2021–2 April 2022. Adjusted hazard ratio (aHR) with a 95% confidence interval (95% CI). Obs. = complete analysis observations.

Terms			Multivariable Cox Regression aHR (95% CI)		
			MODEL 1 (1815 obs.)	MODEL 2 (1791 obs.)	MODEL 3 (2053 obs.)
**Sex**	**Female**		Reference	Reference	Reference
	**Male**		1.24 (0.87; 1.77)	1.23 (0.82; 1.85)	1.09 (0.88; 1.34)
**Age** (years)	**<41**		Reference	Reference	Reference
	**41–55**		1.00 (0.56; 1.76)	2.59 (1.39; 4.83)	0.97 (0.72; 1.32)
	**56+**		1.07 (0.61; 1.87)	1.48 (0.75; 2.92)	0.63 (0.45; 0.87)
**Occupation**	**Clerks**		Reference	Reference	Reference
	**Academic staff**		1.51 (0.63; 3.61)	0.85 (0.34; 2.14)	0.79 (0.48; 1.28)
	**PhD students**		1.32 (0.59; 3.93)	0.73 (0.31; 1.71)	0.66 (0.42; 1.02)
	**Postgraduate medical trainees**		2.67 (0.88; 8.06)	2.87 (0.77; 10.65)	2.16 (1.04; 4.48)
	**Short-term contractors**	**Health sector**	1.13 (0.42; 3.01)	1.34 (0.44; 4.02)	1.51 (0.77; 2.96)
		**Other**	3.95^8^	2.31^8^	0.41 (0.13; 1.23)
**Department**	**Administrative & technical support**		Reference	Reference	Reference
	**Physics**		0.50 (0.13; 1.90)	1.94 (0.66; 5.64)	1.49 (0.77; 2.86)
	**Engineering and Architecture**		0.52 (0.18; 1.47)	0.48 (0.14; 1.66)	1.45 (0.88; 2.40)
	**Mathematics and Geosciences**		0.54 (0.16; 1.75)	0.89 (0.26; 3.04)	1.44 (0.80; 2.58)
	**Chemical and Pharmaceutical Sciences**		0.26 (0.03; 2.09)	1.08 (0.29; 3.97)	1.28 (0.60; 2.70)
	**Life Sciences**		0.97 (0.39; 2.43)	0.50 (0.13; 2.02)	1.10 (0.60; 2.01)
	**Economics, Business and Statistics**		1.78^−20^	0.90 (0.25; 3.31)	0.84 (0.36; 1.97)
	**Law, Language and Interpreting Studies**		0.84 (0.25; 2.82)	0.40 (0.05; 3.25)	1.06 (0.48; 2.35)
	**Political and Social Sciences**		0.83 (0.17; 4.06)	0.73 (0.14; 3.78)	0.26 (0.04; 1.98)
	**Human Sciences**		0.60 (0.18; 2.04)	2.39^−16^	1.30 (0.67; 2.51)
	**Medical, Surgical, Health Sciences**		1.67 (0.69; 4.02)	1.09 (0.39; 3.05)	1.05 (0.56; 1.98)
	**Not specified**		3.73^−9^ (1.03^−9^; 1.35^−8^)	7.00^−9^ (1.52^−9^; 3.22^−8^)	3.99 (1.54; 10.32)
**N. doses of COVID-19 vaccine**	**0**			Reference	Reference
	**1**			1.86 (0.62; 5.56)	0.89 (0.27; 2.92)
	**2**			1.53 (0.90; 2.60)	1.50 (0.98; 2.29)
	**3**			0.10 (0.06; 0.16)	0.37 (0.27; 0.52)

**Table 6:** Multiple Cox proportional regression model contrasting the risk of SARS-CoV-2 infection with heterologous (two doses of Vaxzevria, followed by one dose of an m-RNA vaccine) versus homologous immunization (three doses of COVID-19 m-RNA vaccines) during the Omicron transmission period (1 December 2021–2 April 2022). Hazard ratio (HR) with a 95% confidence interval (95% CI). All Cox regression models fitted onto 1752 complete case analysis observations. Dpt. = University Department.

Terms			Crude Risk	Adjusted Risk				
				Sex	Sex + Age	Sex + Age + Job Task	Sex + Age + Dpt	Sex + Age + Job Task + Dpt
			HR (95% CI)	aHR (95% CI)	aHR (95% CI)	aHR (95% CI)	aHR (95% CI)	aHR (95% CI)
**Booster dose**		**Heterologous**	Reference	Reference	Reference	Reference	Reference	Reference
		**Homologous**	1.76 (1.37; 2.27)	1.76 (1.36; 2.27)	1.54 (1.18; 2.02)	0.91 (0.64; 1.29)	0.87 (0.61: 1.24)	0.92 (0.62; 1.35)
**Age (years)**			**<41**		Reference	Reference	Reference	Reference
			**56+**		0.49 (0.35; 0.67)	0.64 (0.43; 0.94)	0.51 (0.36; 0.70)	0.66 (0.44; 0.98)
**Job task**	**Administrative clerks**					Reference		Reference
	**Postgraduate medical trainees**					3.49 (2.05; 5.92)		3.11 (1.28; 7.52)
	**Contractors in health sector**					2.09 (1.33; 3.28)		
**Dpt**	**Administrative/technical**						Reference	
	**Medical/Surgical/Health**						2.41 (1.63; 3.58)	
	**Not specified**						2.21 (1.16; 4.19)	5.26 1.76; 15.70)

## Data Availability

The data generated and analyzed during the current study are not publicly available, since they were purposively collected by the authors for the present study, but they are available from the corresponding author on reasonable request.
